# Proteome changes of lungs artificially infected with H-PRRSV and N-PRRSV by two-dimensional fluorescence difference gel electrophoresis

**DOI:** 10.1186/1743-422X-7-107

**Published:** 2010-05-26

**Authors:** Shuqi Xiao, Qiwei Wang, Jianyu Jia, Peiqing Cong, Delin Mo, Xiangchun Yu, Limei Qin, Anning Li, Yuna Niu, Kongju Zhu, Xiaoying Wang, Xiaohong Liu, Yaosheng Chen

**Affiliations:** 1 State Key Laboratory of Biocontrol, School of Life Sciences, Sun Yat-sen University, Guangzhou 510006, China

## Abstract

**Background:**

Porcine reproductive and respiratory syndrome with PRRS virus (PRRSV) infection, which causes significant economic losses annually, is one of the most economically important diseases affecting swine industry worldwide. In 2006 and 2007, a large-scale outbreak of highly pathogenic porcine reproductive and respiratory syndrome (PRRS) happened in China and Vietnam. However little data is available on global host response to PRRSV infection at the protein level, and similar approaches looking at mRNA is problematic since mRNA levels do not necessarily predict protein levels. In order to improve the knowledge of host response and viral pathogenesis of highly virulent Chinese-type PRRSV (H-PRRSV) and Non-high-pathogenic North American-type PRRSV strains (N-PRRSV), we analyzed the protein expression changes of H-PRRSV and N-PRRSV infected lungs compared with those of uninfected negative control, and identified a series of proteins related to host response and viral pathogenesis.

**Results:**

According to differential proteomes of porcine lungs infected with H-PRRSV, N-PRRSV and uninfected negative control at different time points using two-dimensional fluorescence difference gel electrophoresis (2D-DIGE) and mass spectrometry identification, 45 differentially expressed proteins (DEPs) were identified. These proteins were mostly related to cytoskeleton, stress response and oxidation reduction or metabolism. In the protein interaction network constructed based on DEPs from lungs infected with H-PRRSV, HSPA8, ARHGAP29 and NDUFS1 belonged to the most central proteins, whereas DDAH2, HSPB1 and FLNA corresponded to the most central proteins in those of N-PRRSV infected.

**Conclusions:**

Our study is the first attempt to provide the complex picture of pulmonary protein expression during H-PRRSV and N-PRRSV infection under the in vivo environment using 2D-DIGE technology and bioinformatics tools, provides large scale valuable information for better understanding host proteins-virus interactions of these two PRRSV strains.

## Background

Porcine reproductive and respiratory syndrome (PRRS) has become one of the most economically important diseases affecting swine industry worldwide, causing significant economic losses each year[1]. The disease was initially found in North America in 1987[2], Europe in 1990[3], China in 1996[4], and Sweden in 2007[5]. PRRS results in both reproductive failure in pregnant sows and respiratory distress in young pigs, such as late-term abortions and stillbirths, premature farrowing, mummified pigs, interstitial pneumonia, respiratory difficulties, high mortality in piglets, and so on[2]. The etiologic agent of PRRS is PRRS virus (PRRSV), a small enveloped, linear, single, positive-stranded RNA virus, which is a member of the family Arteriviridae which includes lactate dehydrogenase-elevating virus (LDV), equine arteritis virus (EAV), and simian hemorrhagic fever virus (SHFV) and enters in the newly established order of the Nidovirales together with the Coronaviridae and Roniviridae family[6]. According to genomic and antigenic differences, and different geographic origins, PRRSV can be classified into two major genotypes: the North American type (NA PRRSV) and the European type (EU PRRSV)[7,8]. To date, PRRSV strains characterized in China are all the NA PRRSV. In 2006 and 2007, the unparalleled large-scale outbreaks of highly pathogenic PRRS (H-PRRS) affected over 2,000,000 pigs with about 400,000 fatal cases and at least 65,000 pigs in China[9,10] and Vietnam[10,11], respectively, which posed great concern to the global swine industry and to public health. Studies showed that highly virulent Chinese-type PRRSV (H-PRRSV) is the major causative pathogen of H-PRRS[9].

Preliminary results indicated that PRRSV strongly modulates the host's immune responses. Studies showed that the virus was able to inhibit IFN-a responses in the lungs of pigs, and may significantly increase IL-10, IFN-γ, IFN-β, TNF-α, MX1, RHIV1, and USP mRNA expression[12-15]. However, mRNA abundance is not always consistent with the protein level[16], factors including post-transcriptional changes in mRNA, post-translational modifications of proteins and microRNAs, which regulate the conversion of mRNAs to proteins[17]. Therefore, information about proteins changes during PRRSV infection may be crucial for us to understand host response to virus and viral pathogenesis. Proteomics analysis is a powerful tool for global evaluation of protein expression, and gaining better insight into the host response to PRRSV. Proteomics has been initially used successfully in the pathogenesis studies, biomarker identification, and protein-protein interaction studies in human disease processes[18]. This approach has been recently applied in animal viral diseases, such as the differential proteomes of chicken embryo fibroblasts after Infectious bursal disease virus (IBDV) infection[19], the cellular changes in Vero cells infected with African swine fever virus[20], proteomic alteration of PK-15 cells after infection by classical swine fever virus[21]. Haiming Zhang and his colleagues identified 23 cellular proteins of PAMs infected with PRRSV in vitro with significant alteration in different courses post-infection by proteomic approaches. Heat shock 27 kDa protein (HSP27) and superoxide dismutase 2 (SOD2), involved in stress response or ubiquitin-proteasome pathway, were observed to be up-regulated[22]. The primary cellular target of PRRSV is the alveolar macrophage of lung and PRRSV infection results in widespread apoptosis in the lungs and lymphoid tissues [23]. However, host response to highly virulent Chinese-type PRRSV (H-PRRSV) and non-high-pathogenic North American-type PRRSV strains (N-PRRSV) in porcine lungs has not been analyzed by comparative proteomics profiling which may be very critical to better understand novel characters of H-PRRSV.

Two-dimensional gel electrophoresis (2-DE) is widely used for proteomics research. However, integral variation and excessive time/labor costs have been common problems with standard 2-DE[24].Two-dimensional fluorescence difference gel electrophoresis (2D-DIGE) technology has recently been implemented as a quantitative alternative to conventional 2-DE [25]. 2D-DIGE enables the labeling of 2-3 samples with different dyes (Cy2, Cy3 and Cy5) and electrophoresis of all the samples on the same 2D gel, reducing spot pattern variability and the number of gels in an experiment and yielding simple and accurate spot matching[17]. Besides, an internal standard labeled with Cy2 dye is used in every gel that reduces inter-gel variation and false positives and increases the robustness of statistical analysis. 2D-DIGE system allows accurate detection of minor differences of protein expression across multiple samples simultaneously with statistical confidence by using the DeCyder software. The comparison of spot intensities using the 2D-DIGE approach and DeCyder software is more objective than the conventional approach based on the comparison of the brightness of gel images obtained by conventional staining and thus has been applied to proteomics studies[24,26]. Using 2D-DIGE followed by MALDI-TOF or MALDI-TOF/TOF identification and bioinformatics methods, we conducted an extensive analysis of proteomes in H-PRRSV and N-PRRSV infected lungs compared with uninfected negative control lungs. In this manuscript we discuss host response to these two viruses through the altered proteins which were identified by comparative analysis of proteomes.

## Results

### Animal model construction

**Figure 1:**
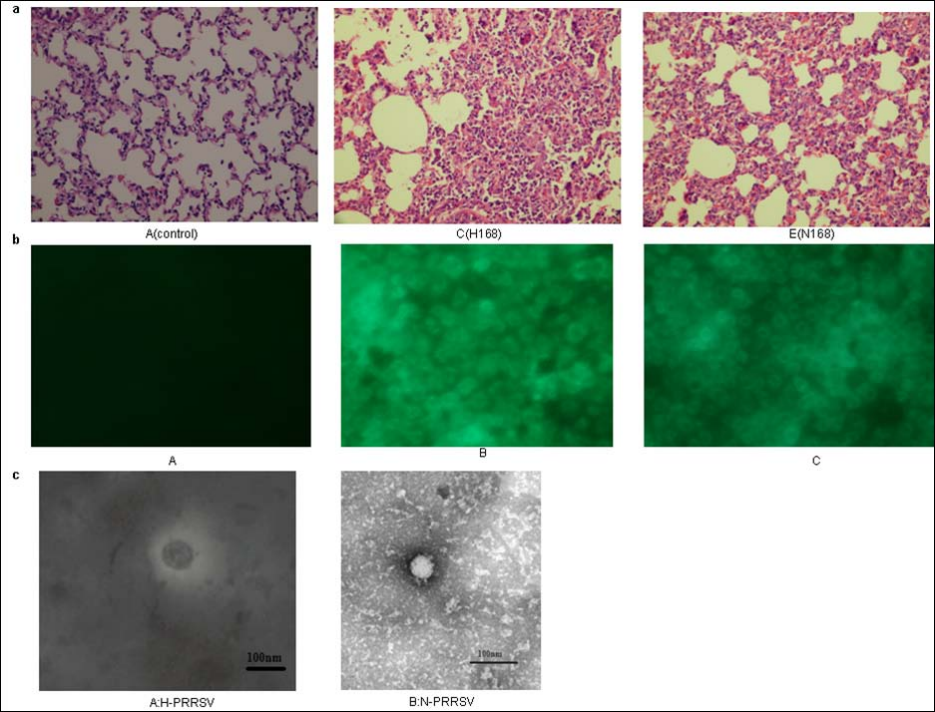
**Identification of lungs infected with H-PRRSV and N-PRRSV**. Lungs of uninfected negative control and experimentally infected pigs were processed routinely for haematoxylin and eosin (H&E) staining and were re-isolated of H-PRRSV and N-PRRSV viruses and then were identified by IFA and EM. Histopathology examination showed an interstitial pneumonia and emphysema in the lungs with thickening of the alveolar septa accompanied with infiltration of mononuclear cells from both H-PRRSV affected pigs and N-PRRSV affected pigs compared to the lungs of negative control pigs. Viral re-isolates were successfully recovered from lungs of the infected pigs, but not from those of uninfected negative control pigs. Specific immunofluorescence and PRRSV particles in MARC-145 cells infected with re-isolated either H-PRRSV or N-PRRSV was observed by IFA and EM, respectively, but not from those of uninfected negative control group. a. Representative images of HE stained lungs sections from H-PRRSV infected(C), N-PRRSV infected(E), and uninfected negative control (A), original magnifications: ×40.; b. Assessment of H-PRRSV(B) or N-PRRSV(C) re-isolated infected MARC-145 cells or negative control(A) by IFA staining at 48 h; c. H-PRRSV particle(A) and N-PRRSV particle(B) under the electron microscopy (EM).

After infection, both H-PRRSV affected pigs and N-PRRSV affected pigs exhibited common clinical symptoms within 3-7 days, including anorexia, rough hair coats, dyspnoea, reddening of skin, oedema of the eyelids, conjunctivitis, mild diarrhoea, shivering, lamping, etc. However, the body temperatures of pigs inoculated with H-PRRSv and N-PRRSV are different. The results are showed as mean ± s.e. H-PRRSV affected pigs exhibited persistently a higher body temperature (41.37 ± 0.23°C) than those N-PRRSV affected (40.43 ± 0.076°C) from 3d pi to 7d pi. Pigs in the uninfected negative control group did not show any obvious changes in body temperature (39.77 ± 0.042°C) and clinical signs. Histopathology examination showed an interstitial pneumonia and emphysema in lungs with thickening of alveolar septa accompanied with infiltration of mononuclear cells from both H-PRRSV affected pigs and N-PRRSV affected pigs compared to lungs of uninfected negative control pigs (Figure 1a). Lungs from all H-PRRSV and N-PRRSV affected pigs were positive for PRRSV by RT-PCR (data not shown). Control pigs lungs were negative for PRRSV by RT-PCR. Subsequently, viral re-isolates were successfully recovered from the infected pigs and confirmed by RT-PCR detection, IFA, and EM. The sequences of NSP2 gene from the re-isolated virus were completely identical with those of the inoculated virus by sequencing. Specific immunofluorescence (Figure 1b) and PRRSV particles (Figure 1c) in MARC-145 cells infected with re-isolated either H-PRRSV or N-PRRSV was observed by IFA and EM, respectively, but not from those of uninfected negative control group.

### Analysis of Differentially Expressed Proteins by 2D-DIGE

**Figure 2:**
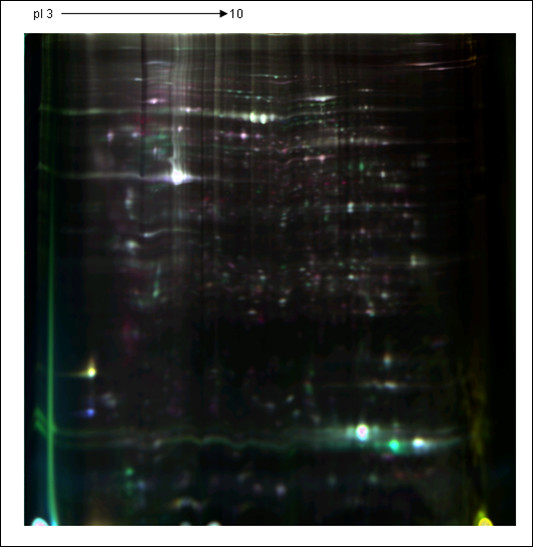
**A representative 2D-DIGE picture of an overlay of three dye scan**. Proteins were extracted as described and separated in pH 3-10 of 13 cm IPG strips for the first dimension and 12.5% acrylamide for the second dimension. Image was acquired on a Typhoon 9400 scanner. Dots represent spots detected by Decyder software. Cy2 (blue) image of proteins from an internal standard is the pool of all the samples, Cy3 (green) image of proteins from control1, and Cy5 (red) image of proteins from H168_2.

A representative picture of an overlay of three dye scan-images Cy2, Cy3, and Cy5 between samples was showed in Figure 2. The estimated number of protein spots was set at 1600 in the pH range of 3-10. From this initial point, the software detected 1465.8 ± 105.75 spots (mean ± SD, n = 8 gel images). 2D-DIGE analyses rendered 14 and 26 spots that exhibited statistically significant expression changes across H-PRRSV infected groups (uninfected negative control; 96 h post H-PRRSV-inoculation, H96; 168 h post H-PRRSV-inoculation, H168) and N-PRRSV infected groups (uninfected negative control; 96 h post N-PRRSV-inoculation, N96; 168 h post N-PRRSV-inoculation, N168), respectively (ONE-ANOVA, p < 0.01). 19 and 8 protein spots differentially expressed between different conditions (H96 vs N96, and H168 vs N168) were obtained by Independent Student's t-test contrast (Average Ratio > 1.5 or Average Ratio < -1.5, p < 0.05).

### Identification of Differentially Expressed Proteins

As shown in Tables 1, 2 and 3, 48 differentially expressed spots were successfully identified as 45 proteins. The majority of spots contained only single proteins but in some cases multiple spots flagged the same protein identity, such as three of spots (460, 481, and 484) were all identified as lamin C, thus indicating the existence of post-translational modifications or different isoforms.

**Table 1:** Different expression of proteins between H-PRRSV (H96, H168) inoculated lungs and control identified by MALDI-TOF or MALDI-TOF/TOF.

Master no.^a^	Accession no.^b^	Human protein (Abbr.)	p Value^c^	Mr (Da)	pI	Protein score^d^	Sequence Coverage (%)^e^
187	gi|134085736	ARHGAP29	0.0044	107466	5.65	69	13
342	gi|136192	TF	0.0047	78971	6.93	71	15
352	gi|74005206	NDUFS1	0.0045	81056	6.1	71	15
371	gi|126309857	HSPA8	0.0055	54213	5.74	97	25
381	gi|194037328	KRT79	0.0087	48266	6.07	67	18
467	gi|68317041	STIP1	0.0029	32224	8.86	106	58
507	gi|231467	AHSG	0.0036	39199	5.5	73	10
552	gi|82822840	DPYSL2	0.0018	31025	8.07	84	11
690	gi|2724046	ACTG1	0.00037	36099	5.65	112	10
882	gi|27807289	ANXA2	0.0089	38873	6.92	73	6
885	gi|23706161	IDH3A	0.0014	27981	9.62	165	15
1461	gi|809283 + gi|1709082	HBB	0.0031	16082 + 19200	6.76 + 6.37	102 + 70	60 + 43

a) Master no. is the unique sample spot protein number.

b) Accession is the MASCOT result of MALDI-TOF/TOF searched from the NCBI nr database.

c) The p value of ONE-ANOVA, p < 0.01, or Independent Student's t-test contrast, p < 0.05.

d) Protein score (based on combined MS and MS/MS spectra) and best ion score (based on MS/MS spectra) were from MALDI-TOF/TOF identification.

e) Sequence coverage (%) is the number of amino acids spanned by the assigned peptides divided by the sequence length.

**Table 2:** Different expression of proteins between N-PRRSV (N96, N168) inoculated lungs and control identified by MALDI-TOF or MALDI-TOF/TOF.

Master no.^a^	Accession no.^b^	Human protein (Abbr.)	p Value^c^	Mr(Da)	pI	Protein score^d^	Sequence Coverage (%)^e^
242	gi|74008809	FLNA	0.0025	283130	5.74	123	1
316	gi|5821963	ACO2	0.0088	83137	7.69	110	3
481	gi|66352015	LMNA	0.0021	65189	6.4	89	30
612	gi|194674843	NTF4	0.00044	33968	9.06	72	27
614	gi|2624886	ALDH2	0.0045	54859	6.05	147	6
616	gi|40426087	LAP3	0.0063	31073	5.64	193	14
638	gi|47685624	ALDH9A1	0.0097	26192	5.43	125	16
656	gi|190360675	FLOT1	0.0089	47554	7.66	74	27
666	gi|126335980	CCDC13	0.0084	83367	7.68	69	19
938	gi|194033965	ANXA1	0.0076	35689	7.16	75	29
1016	gi|149409809	FECH	0.0033	49485	8.48	66	20
1038	gi|87217590	DDAH2	0.0049	23244	5.33	88	39
1173	gi|50916342	HSPB1	0.00092	14268	5.94	73	43
1200	gi|544445	GSTP1	0.0036	23710	8.07	166	13
1250	gi|17892411	PEBP1	0.0019	17055	5.74	149	22
1312	gi|543113	TAGLN	0.0039	19326	6.96	61	11
1316	gi|5031635	CFL1	0.0093	18719	8.22	43	6
1491	gi|21545648	COX5A	0.0026	19379	6.88	82	14

a) Master no. is the unique sample spot protein number.

b) Accession is the MASCOT result of MALDI-TOF/TOF searched from the NCBI nr database.

c) The p value of ONE-ANOVA, p < 0.01, or Independent Student's t-test contrast, p < 0.05.

d) Protein score (based on combined MS and MS/MS spectra) and best ion score (based on MS/MS spectra) were from MALDI-TOF/TOF identification.

e) Sequence coverage (%) is the number of amino acids spanned by the assigned peptides divided by the sequence length.

**Table 3:** Different expression of proteins between H-PRRSV and N-PRRSV (N96/H96, H168/H168) inoculated lungs identified by MALDI-TOF or MALDI-TOF/TOF.

Master no.^a^	Accession no.^b^	Human protein (Abbr)	Average ratio^f^	*p *Value^c^	*M*r (Da)	*p*I	Protein score^d^	Sequence Coverage^e ^(%)
N96/H96								
484	gi|66352015	LMNA	-3.14	0.0075	65189	6.4	76	36
1173	gi|50916342	HSPB1	-2.35	0.00063	14268	5.94	73	43
477	gi|61867592	STIP1	-2.19	0.033	63056	6.02	41	1
604	gi|410689	LAP3	-2.01	0.0092	55996	5.68	66	6
481	gi|66352015	LMNA	-1.99	0.012	65189	6.4	89	30
374	gi|27806351	EZR	-1.92	0.035	68832	6.06	70	1
460	gi|66352015	LMNA	-1.83	0.035	65189	6.4	68	26
1303	gi|40423533	AP3S2	-1.77	0.034	29516	11.16	100	37
1415	gi|15082144	SOD1	-1.66	0.047	15408	6.04	78	21
1312	gi|543113	TAGLN	-1.6	0.04	19326	6.96	61	11
520	Gi|194034593	KIAA1468	-1.58	0.00093	120185	5.38	70	9
848	gi|37800811	GPD1L	-1.55	0.029	22781	5.4	90	46
1292	gi|182851479	VIM	1.6	0.011	18149	4.7	137	72
921	gi|54020966	ANXA2	1.63	0.045	38795	6.49	52	7
942	gi|148747594	RPLP0	1.63	0.026	34508	5.71	159	18
616	gi|40426087	LAP3	1.74	0.0073	31073	5.64	193	14
1428	gi|89886167	FABP5	1.82	0.018	15485	6.6	100	31
N168/H168								
1235	gi|959814	FUT1	-1.59	0.02	15873	9.52	184	35
1171	gi|27806479	PKP1	1.7	0.008	81498	9.18	68	17
1519	gi|6843240	HBA2	2.09	0.00028	13025	8.81	182	25
1506	gi|6843240	HBA2	2.21	0.01	13025	8.81	220	30

a) Master no. is the unique sample spot protein number.

b) Accession is the MASCOT result of MALDI-TOF/TOF searched from the NCBI nr database.

c) The p value of ONE-ANOVA, p < 0.01, or Independent Student's t-test contrast, p < 0.05.

d) Protein score (based on combined MS and MS/MS spectra) and best ion score (based on MS/MS spectra) were from MALDI-TOF/TOF identification.

e) Sequence coverage (%) is the number of amino acids spanned by the assigned peptides divided by the sequence length.

f) Average ratios were calculated considering 6 replica gels and were calculated using Decyder software as the fold -change between normalized spot volume between N-PRRSV-infected lungs (N96 or N168) and H-PRRSV-infected lungs (H96 or H168) homogenates (Independent Student's t-test was based on the log of the ratio between N96 and H96, or between N168 and H168).

### GO enrichment and pathway analysis

These identified proteins were sorted by the enrichment of GO categories (Additional file 1). 12 and 18 proteins were revealed as differentially expressed across H-PRRSV infected groups (uninfected negative control, H96, H168) and N-PRRSV infected groups (uninfected negative control, N96, N168), respectively (Tables 1, 2 and Additional file 2). The high-enrichment GOs targeted by H-PRRSV infected groups proteins were ferric iron transport, positive regulation of myelination, response to organic cyclic substance, pinocytosis, nitric oxide transport, positive regulation of phagocytosis, regulation of inflammatory response, acute-phase response, response to stress, etc (Additional file 2). In contrast, significant GOs corresponding to N-PRRSV infected groups proteins appeared to be actin crosslink formation, ameboidal cell migration, cytoplasmic sequestering of protein, T cell proliferation, anti-apoptosis, oxidation reduction, etc (Additional file 2). 19 proteins were revealed as differentially expressed between H-PRRSV infected lungs and N-PRRSV infected lungs (Table 3). The high-enrichment GOs targeted by N-PRRSV vs H-PRRSV infected groups proteins were ameboidal cell migration, myelin maintenance in the peripheral nervous system, myeloid cell homeostasis, intermediate filament-based process, negative regulation of cholesterol biosynthetic process, regulation of T cell differentiation in the thymus, T cell proliferation, response to superoxide, response to heat, activation of MAPK activity, response to stress, etc (Additional file 2). Pathway analysis was mainly based on the KEGG, BioCarta and REATOME bioinformatics database. These identified proteins were sorted by the enrichment of signaling pathway categories. (Additional file 3). The significant signaling pathways of these identified proteins H-PRRSV infected groups include cell communication, the role of FYVE-finger proteins in vesicle transport, hemoglobin's chaperone, citrate cycle (TCA cycle), pathogenic Escherichia coli infection, vibrio cholerae infection, adherens junction, membrane trafficking,and antigen processing and presentation, etc (Additional file 3). In contrast, significant signaling pathways corresponding to N-PRRSV infected groups proteins appeared to be ascorbate and aldarate metabolism, 3-Chloroacrylic acid degradation, limonene and pinene degradation, beta-Alanine metabolism, urea cycle and metabolism of amino groups, histidine metabolism, fatty acid metabolism, MAPK signaling pathway, glutathione metabolism, stress induction of HSP regulation, induction of apoptosis through DR3 and DR4/5 death receptors, FAS signaling pathway (CD95), signal transduction through IL1R, TNFR1 signaling pathway, p38 MAPK signaling pathway, and caspase cascade in apoptosis, etc (Additional file 3). Significant signaling pathways corresponding to N-PRRSV versus H-PRRSV infected groups proteins include apoptosis, cardiac protection against reactive oxygen species (ROS), cell communication, cystic fibrosis transmembrane conductance regulator (CFTR) and beta 2 adrenergic receptor (b2AR) pathway, free radical induced apoptosis, glycosphingolipid biosynthesis-lactoseries, stress induction of HSP Regulation, MAPK signaling pathway, induction of apoptosis through DR3 and DR4/5 death receptors, FAS signaling pathway (CD95), TNFR1 signaling pathway, and p38 MAPK signaling pathway, etc (Additional file 3).

### Construction of the protein-protein interaction network

**Figure 3:**
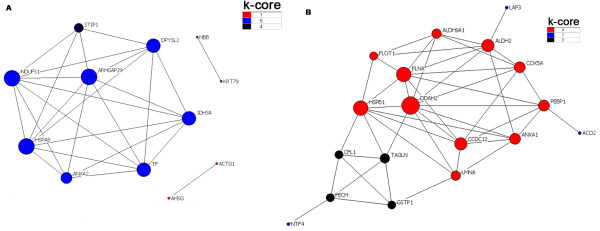
**Graph of the protein interaction network of identified proteins**. The protein interaction network was constructed from the identified proteins according their properties and expression level in differential samples. A) graph of the protein interaction network from identified proteins of H-PRRSV-infected lungs, HSP70, NDUFS1,and GMIP show the highest degree (7) belonging to the most central protein, therefore they might be of great importance to the protein-protein interaction network; B) graph of the protein interaction network from identified proteins of N-PRRSV-infected lungs, DDAH2 with the highest degree (10) followed by another two proteins (HSP27(HSPB1) and FLNA) with degree(8), tend to be more essential than non-central proteins in modular organization of the protein-protein interaction network.

As shown in Figure 3A, three proteins (HSPA8 (HSP70), NDUFS1,and ARHGAP29) show the highest degree(7) belonging to the most central protein followed by another three proteins (TF, IDH3A, and DPYSL2) with degree (6), therefore they might be of great importance to the protein-protein interaction network constructed based on the differentially expressed proteins from lungs H-PRRSV infected. In contrast, as shown in Figure 3B, the most central protein corresponding to those of N-PRRSV infected is DDAH2 with the highest degree (10) followed by another two proteins (HSPB1 (HSP27) and FLNA) with degree (8), these proteins tend to be more essential than non-central proteins in modular organization of the protein-protein interaction network.

### Protein validation by Western blot and Immunohistochemistry

**Figure 4:**
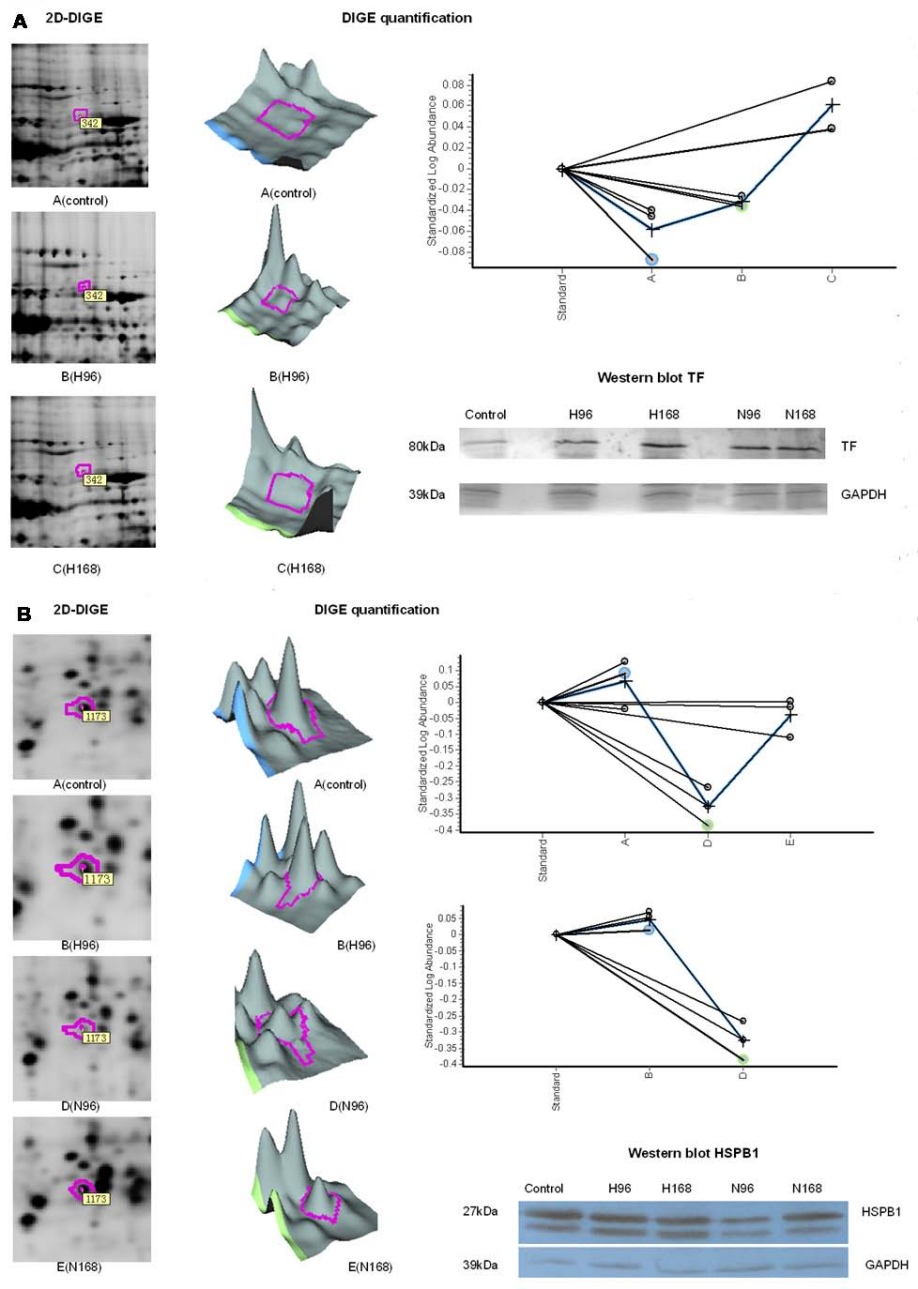
**Expression analyses of selected proteins using DeCyder software and western blot validation**. A) Representative 2D-DIGE image, quantification, and western blot confirmation of TF in H-PRRSV infected pigs. The standard abundance of the different spots (y-axis) is also shown for the three different experimental conditions: A (control), B (H96), C (H168) (x-axis). Equal amounts of total protein, as shown for GAPDH, were loaded for Western blotting analysis; B) Representative 2D-DIGE image, quantification, and western blot confirmation of HSPB1 in N-PRRSV infected pigs and those between N-PRRSV vs. H-PRRSV. The standard abundance of the different spots (y-axis) is also shown for different experimental conditions: A (control), D (N96), E (N168), B (H96) (x-axis). Equal amounts of total protein, as shown for GAPDH, were loaded for Western blotting analysis.

**Figure 5:**
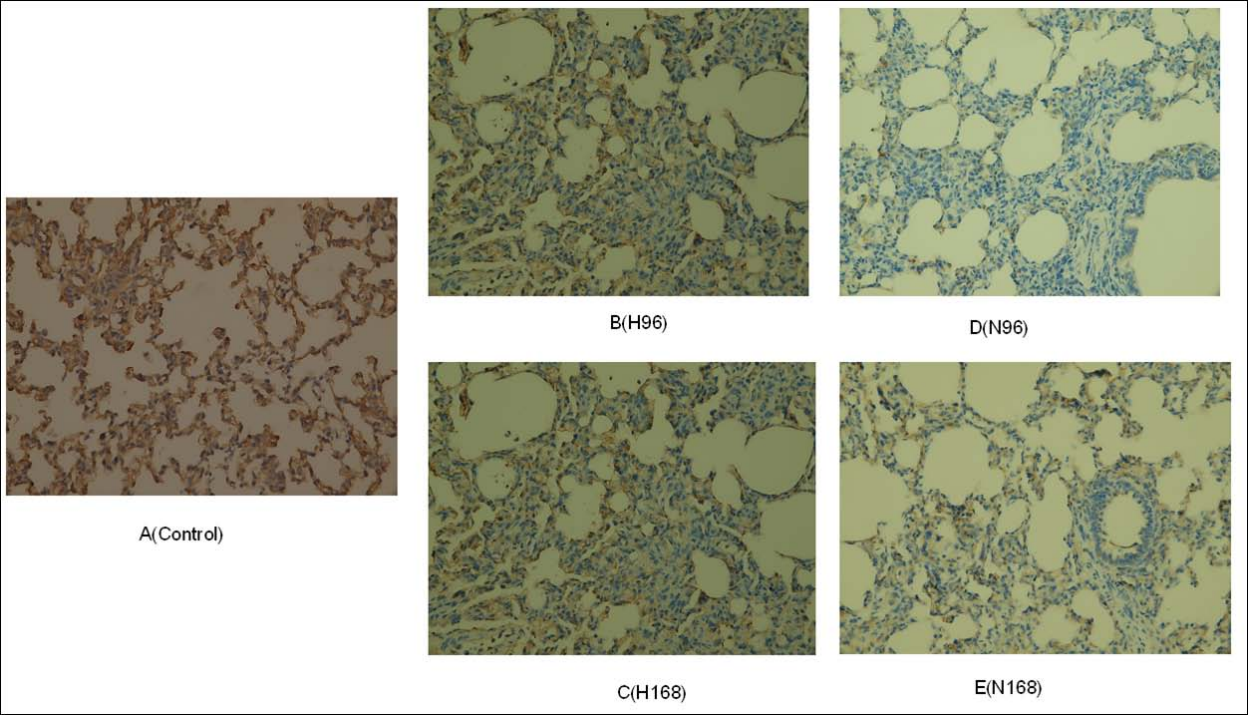
**Immunohistochemistry validation of HSPB1**. The expression pattern of HSPB1 in lungs infected with H-PRRSV and N-PRRSV was investigated by immunohistochemistry. Uninfected negative control lungs, lungs infected with H-PRRSV (H96 and H168), and lungs infected with N-PRRSV (N96 and N168) were stained with anti-HSP27 antibodies. Original magnifications: ×40.

As shown in Figure 4A, TF was slightly up-regulated in lungs H-PRRSV affected at 96 h pi and then strongly up-regulated in those at 168 h pi as compared to uninfected negative control lungs. HSPB1 was strongly down-regulated in lungs N-PRRSV affected at 96 h pi as compared to uninfected negative control lungs and then slightly up-regulated in those at 168 h pi as compared to those at 96 h pi. The results were consistent with the expression changes shown by the 2D-DIGE analysis (Figure 4A and 4B). Meanwhile, to further confirm the differential expression observed in our 2D-DIGE screening, immunohistochemistry (IH) staining of HSPB1 was also performed on paraffin sections. As shown in Figure 5, the result of IH agreed with the expression changes shown by the 2D-DIGE and western blot analysis.

## Discussion

In this study, we for the first time applied 2D-DIGE-based proteomics to identify the differentially expressed pulmonary proteins of lungs during H-PRRSV and N-PRRSV infection in vivo. In total, of the 48 differentially expressed spots, 45 proteins were identified. The indentified protein functions in diverse biological processes and signaling pathways are formed through GO and pathway analysis. Protein-protein interaction network was constructed based on the correlation relationships between individual proteins across the data of differentially expressed proteins from lungs infected with either H-PRRSV or N-PRRSV. The potential roles of some of these changed proteins in response to H-PRRSV and N-PRRSV infection are discussed as follows in relation with pathogenesis and host antiviral response.

### Alteration of cytoskeleton networks and cell communication

Upon infection, virions or subviral nucleoprotein complexes are transported from the cell surface to the site of viral transcription and replication. Viruses use two strategies for intracellular transport: viral components either hijack the cytoplasmic membrane traffic or they interact directly with the cytoskeletal transport machinery[27]. In this study, eight proteins involved in cytoskeleton networks and cell communication have altered. The changes in actin gamma 1(ACTG1), and keratin 79 were detected in H-PRRSV infected lungs, whereas the change of filamin A(FLNA), lamin A/C (LMNA), annexin A1 (ANXA1) and cofilin 1 (CFL1) were detected in N-PRRSV infected lungs. Moreover, vimentin of N-PRRSV-infected (N96) lungs was up-regulated compared to those of H-PRRSV-infected (H96), whereas ezrin and LMNA was down-regulated. These results showed that H-PRRSV and N-PRRSV have to manipulated and utilize host cytoskeleton to promote viral infection like many other viruses[28,29].

FLNA is an actin-binding and signal mediator scaffolding protein that crosslinks actin filaments and links actin filaments to membrane glycoproteins. The encoded protein is involved in remodeling the cytoskeleton to effect changes in cell shape and migration. FLNA is to be as an adaptor protein that links HIV-1 receptors to the actin cytoskeleton remodeling machinery, which may facilitate virus infection[30]. On the other hand, FLNA plays a pivotal role in FcgammaRI surface expression via retention of FcgammaRI from a default lysosomal pathway[31]. FLNA positively regulates I-KappaB kinase/NF-kappaB cascade [32] and transcription factor import into nucleus[33]. In our present study, this protein was strongly down-regulated in N-PRRSV affected lungs at 96 h p.i as compared to uninfected negative control lungs and then slightly up-regulated in those at 168 h p.i as compared to those at 96 h p.i. This phenomenon may explain that N-PRRSV manipulate and utilize the adaptor protein, FLNA, to promote viral infection.

### Response to stress

The quantities of three proteins related to stress response were found to have been modified in either H-PRRSV-infected lungs or N-PRRSV-infected lungs, including heat shock 70 kDa protein 8 (HSPA8, Hsp70), heat shock 27 kDa protein 1 (HSPB1), and stress-induced-phosphoprotein 1. HSPA8 belongs to the heat shock protein 70 family which is highly abundant cytosolic and nuclear molecular chaperones that play essential roles in various aspects of protein homeostasis, controlling the biological activity of folded regulatory proteins, disassembly of clathrin-coated vesicles, viral capsids and the nucleoprotein complex, intracellular vesicle trafficking and sorting, antigen processing and presentation, MAPK signal transduction, cell cycle regulation, differentiation and programmed cell death and nuclear transport. Over expression of hsp70 with a herpes viral amplicon vector protected cultured hippocampal rat neurons from gp120 of HIV neurotoxicity [34], hsp70 was also able to prevent the WNV capsid protein's cytotoxic effects [35], suggesting a protective cell function for this molecular chaperone against viral infection. The exposure of permissive CD4+ cells to HIV-1 gp120 increases the synthesis and nuclear translocation of 70 kDa heat shock protein. Hsp70 facilitates nuclear import of HIV-1 preintegration complexes by stimulating the binding of HIV-1 Matrix to karyopherin alpha. Over-expression of Hsp70 by WNV infection, hepatitis C virus (HCV) infection[36], and TBSV infection[37] suggests that it involves in the pathogenesis of those viruses. In the present study, HSPA8 was up-regulated continuously after H-PRRSV infection. Moreover, in the protein-protein interaction network constructed based on the differentially expressed proteins from lungs H-PRRSV infection, HSPA8 shows the highest degree (7) belonging to the most central protein. The most central protein tends to be more essential than non-central proteins in modular organization of the protein-protein interaction network. These results suggest that Hsp70 might be involved in H-PRRSV pathogenesis and as a specific chaperone, it can protect cell from apoptosis.

Heat shock 27 kDa protein (HSPB1, Hsp27) is a stress-inducible ubiquitous cellular protein that belongs to small HSP families and is involved in cellular protection in response to a variety of stresses such as heat shock, toxicants, and oxidative stress, stress induction of HSP regulation, MAPK signaling pathway, anti-apoptosis, regulation of translational initiation, molecular chaperoning, actin organization and cell motion. Hsp27 regulates Akt activation and cellular apoptosis by mediating interaction between Akt and its upstream activator MK2[38]. Moreover, the phosphorylated Hsp27 binded by caspase-3 prodomain regulates monocyte apoptosis by inhibiting caspase-3 proteolytic activation[39]. Viral infection modulates the regulation of apoptosis in host cells. Up-regulated HSP27 has been found in cells infected with Epstein-Barr virus[40], avian H9N2[41], Afriacan swine fever virus[20], IBDV[19], and PRRSV[42]. But down-regulated HSP27 has been also found in cells infected with classical swine fever virus [21] and IBDV (another HSPB1 protein spot)[19]. In the present study, this protein was strongly down-regulated in N-PRRSV affected lungs at 96 h p.i as compared to uninfected negative control lungs and then slightly up-regulated in those at 168 h p.i as compared to those at 96 h p.i. Moreover, in the protein-protein interaction network constructed based on the differentially expressed proteins from lungs N-PRRSV infected, Hsp27 shows the very highly degree (8) belonging to the central protein. Some evidences indicate that human cells infected with mumps virus become susceptible to apoptosis caused by extracellular stresses. The infected cells failed to acquire resistance to apoptotic stimuli (thermotolerance) after exposure to these mild stresses. The induction of Hsp27 was dramatically suppressed after mumps virus infection through the destruction of STAT-1[43]. Based on these data, Hsp27 might be involved in N-PRRSV pathogenesis, and the lack of thermotolerance should allow the infected cells to be eliminated by apoptosis and might be a host defense against viral infection.

### Oxidation reduction and metabolism

Four differentially expressed proteins of interest associated with oxidation reduction and metabolism were found, including Isocitrate dehydrogenase 3 (NAD+) alpha (IDH3A), NADH dehydrogenase Fe-S protein 1 (NDUFS1) and Annexin A2 (ANXA2) in H-PRRSV infected lungs; Glutathione S-transferases P(GST class-pi, GSTP1) in N-PRRSV infected lungs; Superoxide dismutase 1, soluble (SOD1) and Ribosomal protein, large, P0 between H-PRRSV and N-PRRSV infected lungs.

NDUFS1 belongs to the complex I 75 kDa subunit family, playing a very important role in the electron transport from NADH to ubiquinone in the respiratory chain for ATP production. GO analysis in our study also classified NDUFS1 as ATP synthesis coupled electron transport. Previously, studies indicated that HIV-1 infection induced to release ROS through a mitochondrial pathway. In addition, Disruption of electron transport and mitochondrial transmembrane potential, loss of ATP production and promotion of ROS generation were due to cleavage NDUFS1 by caspases. However cells expressing a noncleavable mutant of NDUFS1 sustain mitochondrial transmembrane potential and ATP levels during apoptosis and ROS generation is dampened in response to apoptotic stimuli. All of these indicated that caspase cleavage of NDUFS1 is essential to several changes of mitochondrion during apoptosis[44]. On the other hand, reduced expression of NDUFS1 was found in chronic morphine treated hippocampal and down-regulation of NDUFS1 would decrease of ATP production[45]. Therefore, the continuous increased expression of NDUFS1 in H-PRRSV infected lungs might provide continuous increased substrate for apoptosis and also sustain energy metabolism. This is supported by the previous findings that inhibition of complex I activity would lead to reduction of ATP levels in HIV-infected cells, but ATP synthesis would not be ceased completely[46]. Hence, these results might be mainly implicated in how H-PRRSV influenced host cell energy metabolism during apoptotic cell death. Additionally, the degree of NDUFS1 in the protein network of H-PRRSV infected lungs is seven, which ranked the first. Hence, NDUFS1 located at the most central in the network. This implies that NDUFS1 is likely to be more essential in organization of protein-protein interaction network.

### Apoptotic pathways

Apoptosis of host cells plays an important role in modulating the pathogenesis of many infectious diseases. Dimethylarginine dimethylaminohydrolase 2 (DDAH2) belongs to the dimethylarginine dimethylaminohydrolase (DDAH) gene family and involves in anti-apoptosis, response to unfolded protein, defense response, nitric oxide biosynthetic process, nitric oxide mediated signal transduction, and arginine catabolic process. The encoded enzyme plays an important role in nitric oxide generation by regulating cellular concentrations of methylarginines, which in turn inhibit nitric oxide synthase activity. The recent study has indicated that the activity of DDAH and the expression of DDAH2 (mRNA and protein) was significantly decreased in cobalt chloride (CoCl_2_)-induced apoptosis. In contrast, DDAH2 overexpression inhibited the proapoptotic effects of CoCl2 [47]. CoCl_2 _significantly increased the level of endogenous nitric oxide synthase inhibitor asymmetric dimethylarginine (ADMA), which markedly increased intracellular ROS production and promoted inflammatory responses, resulting in caspase-3-dependent apoptosis. Moreover, exogenous ADMA could directly induce cellular apoptosis via ROS dependent signaling pathway. DDAH is the specific hydrolase of ADMA and plays an important role in the modulation of ADMA level. Various oxidative, LPS, or inflammatory stimuli could directly inactivate the DDAH activity and then significantly decrease the expression of DDAH2 mRNA and protein through a sulfhydryl group in the catalytic region of DDAH [48]. Moreover, expression of DDAH2 was also found to be reduced when comparing lung tissue from pulmonary hypertensive rats and idiopathic pulmonary arterial hypertension (IPAH) patients to corresponding normal lung tissue [49]. DDAH2 localizes to 6p21.3. The region contains a number of genes involved in the immune and inflammatory responses and has been linked with susceptibility to several autoimmune diseases. This localization and its wide expression in immune cells means that DDAH2 has the potential to be a disease-susceptibility gene[50]. DDAH2 was strongly down-regulated in N-PRRSV affected lungs at 96 h p.i as compared to uninfected negative control lungs and then slightly up-regulated in those at 168 h p.i as compared to those at 96 h p.i. Moreover, in the protein-protein interaction network constructed based on the differentially expressed proteins from lungs N-PRRSV infected, DDAH2 shows the highest degree (10) belonging to the most central protein. These results strongly support the importance of DDAH2 in N-PRRSV pathogenesis, and after N-PRRSV infection, expression of DDAH2 in lungs significantly decreased comparing to those in uninfected negative control lungs, which resulted in cell-infected apoptosis, which might be a host defense against viral infection.

### Others

Rho GTPase activating protein 29 (PARG1, ARHGAP29), encoding for a protein-tyrosine phosphatase-associated Rho GTPase activating protein, is involved in signaling by Rho GTPases. Rho GTPases, regulating GTP-GDP cycle, were key signal transducers, mediating growth factor-induced changes to the actin cytoskeleton and activating the phagocyte NADPH oxidase, and participated in a number of cellular processes, such as cell migration, cell survival, transcriptional regulation and vesicle trafficking. This is because they might be able to interact with lots of downstream targets, so that they can coordinately activate several molecular processes required for a particular cellular response. In the present study, we observed that in the protein-protein interaction network constructed based on the differentially expressed proteins from lungs H-PRRSV infected, ARHGAP29 shows the highest degree (7) belonging to the most central protein. It interacted with sever protein of the network, including HSP70, NDUFS1,IDH3A, TF, DPYSL2, ANXA2, and STIP1, which suggests that these proteins could coordinately activate several molecular processes required for a particular cellular immune response. A strong down-regulation of ARHGAP29, by several mechanisms such as deletion and promoter methylation, was found in all mantle cell lymphoma (MCL) samples, which may lead to carcinogenesis through the dysregulation of Rho/Rac/Cdc42-like GTPases[51]. ARHGAP29 was down-regulated in H-PRRSV affected lungs at 96 h p.i as compared to uninfected negative control lungs and then continuously down-regulated in those at 168 h p.i as compared to those at 96 h p.i. Based on these results, it is reasonable to postulate that ARHGAP29 coordinates other proteins together to involve in the pathogenesis of H-PRRSV.

## Conclusion

We analyzed the protein expression changes of H-PRRSV and N-PRRSV infected lungs compared with those of uninfected negative control, and identified a series of proteins related to viral pathogenesis and host response using 2D-DIGE followed by MS identification and bioinformatics methods. Our results showed that following both H-PRRSV and N-PRRSV infection, the significant expression changes in pulmonary proteins were mostly related to cytoskeletal proteins, stress response proteins and proteins involved in oxidation reduction or metabolism. The changed expression of some cytoskeletal proteins could be a strong sign of cytoskeletal reorganization which is essential for viral reproduction and assembly. Besides, protective proteins in response to a variety of virus-induced stresses such as oxidative stress, heat shock and toxicants have been shown to be expressed differentially after either H-PRRSV or N-PRRSV infection. In the protein-protein interaction network constructed based on the differentially expressed proteins from lungs H-PRRSV infected, HSPA8, ARHGAP29, and NDUFS1 showed the highest degree belonging to the most central protein, but DDAH2, HSPB1, and FLNA corresponded to the most central proteins in those of N-PRRSV infected, suggesting differential viral pathogenesis and differential host response to H-PRRSV and N-PRRSV infection. To our knowledge, the study presented here is the first proteomic study using 2D-DIGE and MS to compare the complex picture of pulmonary protein expression during H-PRRSV and N-PRRSV infection.

## Methods

### Experimental animals and tissue collection

All animal procedures were performed according to guidelines developed by the China Council on Animal Care and protocol approved by Animal Care and Use Committee of Guangdong Province, P.R. China.

Fifteen conventionally-reared, healthy 6-week-old, crossbred weaned pigs (Landrace × Yorkshire) were selected from a high-health commercial farm that has historically been free of all major pig diseases, such as PRRSV, porcine circovirus type 2, classical swine fever virus, porcine parvovirus, pseudorabies virus, swine influenza virus and Mycoplasma hyopneumoniae infections. All pigs were PRRSV-seronegative determined by ELISA (HerdChek PRRS 2XR; IDEXX Laboratories) and absence of PRRSV tested by RT-PCR. Pigs were randomly assigned to one uninoculated negative control group and two PRRSV-inoculated groups (H-PRRSV and N-PRRSV respectively, gift from Dr. Zhang Guihong, South China Agricultural University) in the experiment. Six pigs were inoculated with 6 ml viral suspension (4 ml intranasally and 2 ml intramuscularly) of H-PRRSV at a dose of 10^6.0 ^TCID_50 _ml^-1 ^on day 0. Six pigs were inoculated with 6 ml viral suspension (4 ml intranasally and 2 ml intramuscularly) of N-PRRSV at a dose of 10^6.0 ^TCID_50 _ml^-1 ^on day 0. Three negative control pigs were treated similarly with an identical volume of DMEM culture media from uninfected MARC-145 cells 1 day prior to experimental infection, and were immediately necropsied. Two PRRSV-inoculated groups were clinically examined daily and rectal body temperatures were recorded from days -2 to 7 post infection (p.i). Three infected pigs randomly chosen within each group were necropsied at each time point of 96 h p.i and 168 h p.i. Lung samples were collected from control, three pigs at 96 h post H-PRRSV-inoculation (H96), three pigs at 168 h post H-PRRSV-inoculation (H168), three pigs at 96 h post N-PRRSV-inoculation (N96), three pigs at 168 h post N-PRRSV-inoculation (N168) and immediately frozen in liquid nitrogen for proteome analysis or fixed in 10% neutralized buffered formalin for histological processing.

### Virus re-isolation and RT-PCR detection

250 μl of lung tissue homogenate plus 150 μl of DMEM with 75 μg of penicillin and 50 μg of streptomycin per ml were inoculated on MARC-145 cells and incubated for 1.5 h at 37°C with 5% CO_2_. Then, tissue homogenate were removed and DMEM containing 5% FBS was added. Cultures were incubated for 3 days at 37°C in a 5% CO_2 _humidified incubator. Cultures which do not display cytopathic effect (CPE) after three passages were considered negative. And PRRSV induced CPE on MARC-145 was confirmed by the following three methods: 1) indirect immunoflorescent assay (IFA) using positive serum against PRRSV; 2) negative-stain electron microscopy (EM) which applied 4 μl virus suspension to glow-discharged carbon-coated copper grids with a micropipette and stained with 1% (w/v) uranyl acetate; 3) PRRSV-specific RT-PCR using oligonucleotide primers NSP2F(5'-AACACCCAGGCGACTTCA-3') and NSP2R(5'-GCATGTCAACCCTATCCCAC-3') which designed according to the existing 87 base deletion between the H-PRRSV and N- PRRSV in the fixed site in Nsp2 gene and will amplify 787 bp and 874 bp DNA fragment of H-PRRSV and N-PRRSV, respectively.

### Histological examination

Lungs of uninfected negative control and experimentally infected pigs were processed routinely for haematoxylin and eosin (H&E) staining, as described previously[52].

### Protein Extraction

For each sample, ~0.3 g of lung tissue washed with normal saline was trimmend into 3 mm^3 ^slices and then was homogenized on ice in 1 ml DIGE lysis buffer (7 M Urea, 2 M Thiourea, 4% CHAPS, 0.2%IPGbuffer, protease inhibitor mixture) using a DOUNCE homogenizer. After sonication (8 × 10 s pulses on ice, with cooling intervals of 15 s in between) and centrifugation (14,000 rpm for 1 hour) to collect supernatant fluid, protein concentrations were determined using the Bio-Rad Protein Assay (Bio-Rad). Proteins were checked by visualization of Comassie blue stained proteins separated on a 12.5% SDS-PAGE acrylamide gel. Concentration of all samples was adjusted to 5 μg/μl.

### Protein labeling

Equal amounts of proteins from the 15 samples were pooled together as the internal standard. Proteins were minimally labeled according to the manufacturer's instructions (CyDye DIGE fluor minimal labeling kit, GE Healthcare). Briefly, each miminal CyDye was reconstituted in fresh N,N-dimethylforamide (DMF) and a 400 pmol quantity used to label 50 μg of protein at pH 8.5. Cy2 was used to label the pooled internal standard. Cy3 and Cy5 were used to randomly label the uninfected negative control and H-PRRSV-infected or N-PRRSV-infected samples. The labeling reaction was done on ice in the dark for 40 min and the reaction was terminated by addition of 1 μl 10 mM lysine on ice in the dark for 10 min. To minimize system and inherent biological variation, sample multiplexing was also randomized (Table 4) to produce unbiased results.

**Table 4:** 2D-DIGE experimental design*.

Gel	Cy2(blue)	Cy3(green)	Cy5(red)
1	pool	A1 (Control1)	C2 (H168_2)
2	pool	C1 (H168_1)	E3 (N168_3)
3	pool	D2 (N96_2)	B2 (H96_2)
4	pool	B1 (H96_1)	E2 (N168_2)
5	pool	E1 (N168_1)	A1 (Control1)
6	pool	D3 (N96_3)	C3 (H168_3)
7	pool	B3 (H96_3)	A2 (Control2)
8	pool	A3 (Control3)	D1 (N96_1)

*Control and experimental samples (H96, H168, N96, N168) were labeled with either Cy3 or Cy5. Equal amounts of protein lysates from 3 uninfected negative control and 12 experimental samples were pooled as the internal standard, and labeled with Cy2. Each gel was loaded with 50 μg of Cy2-labeled protein pool, 50 μg of Cy3-labeled and 50 μg of Cy5-labeled samples as indicated. Three replicates were used by each experimental condition.

### 2-D gel electrophoresis

Following the labeling reaction, 50 μg of each Cy2, Cy3 and Cy5 labeled samples were mixed. Then the pooled sample of each gel was diluted with rehydration buffer (7 M urea, 2 M thiourea, 2% DTT (w/v), and 1% IPG buffer (v/v)) to 250 μl before Isoelectric Focusing (IEF). Samples were actively rehydrated into 13-cm pH 3-10 non-linear Immobiline DryStrips, placed in a strip holder and focused with an Ettan IPGphor Isoelectric Focusing System (GE Amersham) using a step gradient protocol ranging from 30 to 8000 volts for approximately twenty six hours (30 v 12 hrs, 500 v 1 hr, 1000 v 1 hr, 8000 v 8 hrs, 500 v 4 hrs).

The IPG strips were rehydrated in re-equilibration buffer (8 M urea, 100 mM Tris-HCL (pH6.8), 30% Glycerol, 1% SDS, 45 mg/mL iodoacetamide (to reduce streaking)) for 10 minutes, and then proteins were further separated on the 12.5% homogeneous SDS-PAGE gels (24 cm × 20 cm × 1 mm) casted with low-fluorescence glass plates utilizing Hofer SE 600 (GE Amersham). The SDS-PAGE gels were run at 15 mA/gel for 20 min and then at 30 mA/gel at 15°C until the bromophenol blue dye front reach the bottom of the gel.

### Scanning and image analysis

After 2D-DIGE, scan the gels using a Typhoon 9400 scanner (GE Amersham) at 100 μm resolution, as elaborated in the equipment setup. The Cy2, Cy3, and Cy5 labeled images for each gel were scanned at the excitation/emission wavelengths of 488/520 nm, 532/580 nm, 633/670 nm, respectively. After scanning the three fluorophores for each gel, the images were imported to the DeCyder image analysis software (GE Amersham) for spot detection according to manufacturer's recommendations. Briefly, Differential in gel analysis (DIA) module was used for intra-gel analysis for protein spot detection and for normalization of Cy3 and Cy5 gel images with respect to the Cy2 image. After spot detection, the abundance changes were represented by the normalized volume ratio (Cy3:Cy2 and Cy5:Cy2). Make sure that artifactual spots (dust and others) were removed and that all true protein spots were included, all the protein spots detected were also examined manually. The biological variation analysis (BVA) module was used for inter-gel matching of internal standard and samples across all gels, and performing comparative cross-gel statistical analyses of all spots, based on spot volumes, permitting the detection of differentially expressed spots between experimental conditions (One-way ANOVA, p < 0.01 and Independent Student's t-test, p < 0.05). The protein spot matches were also confirmed manually for all the gels. Protein spots that were differentially expressed in H-PRRSV infected and N-PRRSV infected groups (B/D,C/E) (Independent Student's t-test, Average Ratio > 1.5 or Average Ratio < -1.5, p < 0.05) were marked. Protein spots that were differentially expressed in H-PRRSV infected and uninfected negative control groups (A/B/C) or N-PRRSV infected and uninfected negative control groups (A/D/E) ((One-way ANOVA, p < 0.01) were marked. Satisfying these criteria, a pick list is generated and exported to the software controlling the Ettan robotic spot picker (GE Amersham). Spots were excised with a 3 mm core from the post-stained gel and loaded to a 96-well plate for digestion. Spots in the maps for which the average intensity differed between two appoint groups were selected to be identified by mass spectrometry.

### Protein digestion, mass spectrometry and protein identification

Preparative gels containing 500 μg protein were run to identify interest protein and were stained with Coomassie brilliant blue (CBB). Protein spots of interest were excised from the gel automatically using an Ettan Spot Picker robot (GE Amersham) and destained with 25 mM ammonium bicarbonate, 50% ACN. Gels were then dried completely by vacuum-drying. In-gel digestion was performed with 12.5 ng/L modified sequencing grade trypsin (Promega) in 25 mM ammonium bicarbonate at 4°C for 40 min prior to 20 h at 37°C. To achieve complete peptide recovery, two sequentially extraction steps (5% TFA at 40°C for 1 h and with 2.5% TFA, 50% ACN at 30°C for 1 h) were carried out with the digested samples. The supernatants containing peptides were then collected, and then concentrated and desalted by ZipTips (Millipore, Bedford, MA). Peptides were mixed with equal amounts of matrix solution (α-cyano-4-hydroxy-cinnamic acid (HCCA) in 0.1% TFA, 50% ACN) and immediately loaded on the target plate, and allowed to air-dry at room temperature. MALDI-TOF mass spectrometry and tandem TOF/TOF mass spectrometry analyses were performed on an AutoFlex TOF-TOF LIFT Mass Spectrometer (Bruker Daltonics) according to the manufacturer's instructions. The spectra were acquired in the positive ion reflection mode (accelerating voltage of 20 kV, reflecting voltage of 23 kV) with external calibration (Trypsin_Roche_porcine_Modified) according to the settings given by the manufacturer. Parent mass peaks with mass range of 700-4000 and minimum signal to noise ratio of 15 were picked out for tandem TOF/TOF analysis. The generated mass lists were subsequently sent to MASCOT (Version 2.1, Matrix Science, London, UK) by GPS Explorer software (Version 3.6, Applied Biosystems) for protein identification. Parameters for searches were as follows: National Center for Biotechnology Information non-redundant (NCBInr) database (EST_chordata chordata_20081008 (87827958 sequences; 17755145374 residues)), taxonomy of other mammalia (23009496 sequences); tryptic peptides with max one missed cleavage site; fixed modifications, carbamidomethylation; variable modifications, oxidation; peptide mass tolerance, ± 150 ppm. MASCOT protein scores (based on combined MS and MS/MS spectra) of greater than 65 were considered statistically significant (p < 0.05). The individual MS/MS spectrum with a statistically significant (p < 0.05) ion score (based on MS/MS spectra) were accepted.

### Gene ontology (GO) and pathway enrichment analysis

GO analysis [53] was applied in order to organize differentially expressed proteins into functional classification on the basis of biological process. Pathway analysis [54-56] was mainly based on the Kyoto Encyclopedia of Genes and Genomes (KEGG) and BioCarta and REATOME bioinformatics database. Two-side Fisher's exact test with a multiple testing and χ2 test were used to classify the GO and pathway category. The false discovery rate (FDR) was used to correct the P-value. We chose only GO categories that had a P-value of <0.01 and an FDR of <0.05 and pathway categories that had a P < 0.05. Within the significant category, the enrichment Re was given by:

n_f _: the number of flagged proteins within the particular category;

n: the total number of proteins within the same category;

N_f_: the number of flagged proteins in the protein reference database list;

N: the total number of proteins in the protein reference database list;

### Construction of the protein-protein interaction network[57,58]

Protein-protein interaction network was constructed based on the data of differentially expressed proteins. The matrix of proteins expression values was build up at first, and then Pearson product-moment correlation coefficients were computed. Suppose there are two variables X and Y, which indicate expression value of two proteins respectively in the sample, with means  and  respectively and standard deviations S_X _and S_Y _respectively. The correlation r is calculated as:

The Pearson product-moment correlation coefficients have been applied to quantify the strength of correlation between proteins. And a correlation coefficient of no less than 0.48 was considered as 1 while which less than 0.48 was considered as 0. Protein correlation matrix (PCM) was then to be formed. According to the correlation between proteins, protein-protein interaction network was constructed. Nodes were applied to represent the proteins and interactions between proteins were expressed by straight lines between the nodes. Then each node's degree was calculated. The nodes with more interactions will have higher degrees. In addition, different colors of nodes indicate different values of K-core the proteins have. The "degree" is defined as the number of interactions of a protein with other proteins in the protein network. While the rank is determined as the decreasing ordering of each protein's degree, the first rank which has the highest degree belongs to the most central protein in the network. The most central protein tends to be more essential than non-central proteins in modular organization of the protein-protein interaction network. K cores have been applied for clustering proteins of network. The proteins with degrees of the same or close to were colored identically and different colored proteins were identified as different subnetworks. Therefore, protein-protein interaction network has been divided into several subgraphs and all the proteins in one subgraph belong to same cluster of degrees. A subnetwork was used to identify a group of same colored proteins, which were found to regulate almost same number of other proteins in the network and implied they shared similar biological functions under certain conditions.

### Western blot analysis

Equivalent amounts of total protein (40 μg) were loaded in each lane and were fractionated by electrophoresis on 12% (w/v) SDS-PAGE gels, then transferred onto a PVDF membrane using iBlot™ Dry Blotting System (Invitrogen) and blocked with TBS-T containing 5% BSA at 4°C overnight. The PVDF membrane were probed with a 1:500 dilution of goat anti-pig Transferrin antibody (Bethyl, TX, USA), and at a dilution of 1:200 mouse anti-Heat Shock Protein 25 monoclonal antibody (Chemicon/Millipore, MA, USA). Horseradish peroxidase-conjugated rabbit anti-goat IgG, horseradish peroxidase-conjugated goat anti-mouse IgG or horseradish peroxidase-conjugated goat anti-rabbit IgG at a dilution of 1:4,000 were used as secondary antibodies. The protein bands were visualized using diaminobenzidine (DAB) as the substrate (Boster, Wuhan, China). The same membranes were reblotted with rabbit affinity purified anti-GAPDH antibody (Rockland, PA, USA) at a dilution of 1:1,000 to confirm equal loading.

### Immunohistochemistry analysis

Lung tissues of uninfected negative control and experimentally infected pigs were formalin fixed for immunohistochemistry. Paraffin sections (5 μm) were deparaffinized and rehydrated in in a graded alcohol series, and pretreated with 10 mM sodium citrate (3-10 min, 600 W microwave oven). Nonspecific binding was blocked by incubating the tissue sections with 10% BSA (Sigma) in PBS for 60 min. Immunostaining was performed in a moist chamber at 37°C for 1 h with mouse anti-Heat Shock Protein 25 monoclonal antibody (Chemicon/Millipore, MA, USA) at a dilution of 1:200. Horseradish peroxidase-conjugated goat anti-mouse IgG at a dilution of 1:4,000 was used as secondary antibodies. Immunoreactions were visualized via an avidin-biotin complex, using the Vectastain ABC alkaline phosphatase kit (distributed by CAMON, Wiesbaden, Germany). Fast red/Naphthol Mx (Immunotech, Marseille, France) served as chromogen.

## Abbreviations

ABPs: actin-binding proteins; ACTG1: actin gamma 1; ADMA: asymmetric dimethylarginine; AHSG: alpha2-HS glycoprotein; ALDH2: aldehyde dehydrogenase 2; b2AR: beta 2 adrenergic receptor; CFL1: cofilin 1; CFTR: cystic fibrosis transmembrane conductance regulator; FECH: ferrochelatase; FLNA: filamin A; FLOT1: flotillin 1; HBA: hemoglobin, alpha; HBB: hemoglobin, beta; HCC: hepatocellular carcinoma; JNK: C-jun N-terminal kinase; KRT79: keratin 79.

## Competing interests

The authors declare that they have no competing interests.

## Authors' contributions

SX, QW and JJ conceived and designed the study. SX and QW performed the experiments. SX, QW and JJ analyzed data, and wrote the manuscript. PC, DM, XY, LQ and YN coordinated the study. YC, KZ and XW contributed to the interpretation of the results and took part to the critical revision of the manuscript. All authors read and approved the final manuscript.

## Supplementary Material

Additional file 1 **Function class of identified proteins**. Analysis of identified protein reveals proteins from diverse functional categories. Functional classification of the identified proteins was performed according to GO biological processes. A P-value of < 0.01 and an FDR of <0.05 in the two-side Fisher's exact test were selected as the significant criteria. These identified proteins were sorted by the enrichment of GO categories. A) the GOs targeted by the differentially expressed proteins in H-PRRSV-infected lungs; B) the GOs targeted by the differentially expressed proteins in N-PRRSV-infected lungs; C) the GOs targeted by the differentially expressed proteins between N-PRRSV and H-PRRSV infected lungs. The vertical axis is the GO category and the horizontal axis is the enrichment of GO. Click here for file

Additional file 2 **Different expression of proteins after PRRSV infected depend on time points**. A) Different expression of proteins between H-PRRSV inoculated lungs and control depend on time points; B) Different expression of proteins between N-PRRSV inoculated lungs and control depend on time points. Click here for file

Additional file 3 **Signaling pathways of identified proteins**. Pathway analysis based on the KEGG, BioCarta, and REATOME bioinformatics database. A P-value of <0.05 and an FDR of <0.05 in the two-side Fisher's exact test were selected as the significant criteria. A) significant signaling pathways of these identified proteins H-PRRSV infected groups; B) significant signaling pathways corresponding to N-PRRSV infected groups proteins; C) significant signaling pathways involved in N-PRRSV versus H-PRRSV infected groups proteins. The vertical axis is the pathway category and the horizontal axis is the lgP(log(p Value)) of these significant pathways. Click here for file
