# Supplementary figures and images for: Proteome changes of lungs artificially infected with H-PRRSV and N-PRRSV by two-dimensional fluorescence difference gel electrophoresis
Source: Virol J. 2010 May 26;7:107. doi: 10.1186/1743-422X-7-107 (PMC2887434; doi:10.1186/1743-422X-7-107)

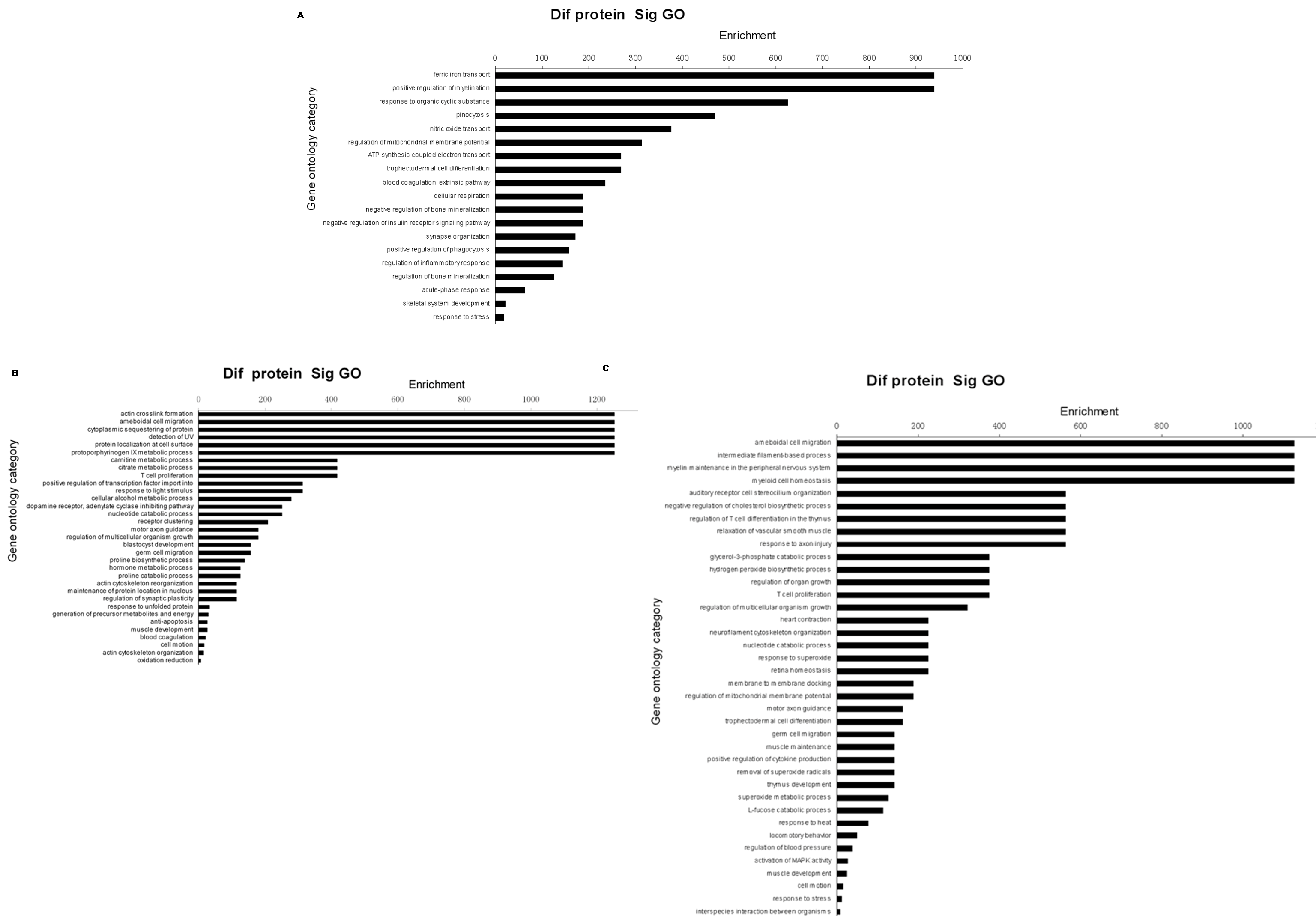

Supplement: Additional file 1 — Function class of identified proteins. Analysis of identified protein reveals proteins from diverse functional categories. Functional classification of the identified proteins was performed according to GO biological processes. A P-value of < 0.01 and an FDR of <0.05 in the two-side Fisher's exact test were selected as the significant criteria. These identified proteins were sorted by the enrichment of GO categories. A) the GOs targeted by the differentially expressed proteins in H-PRRSV-infected lungs; B) the GOs targeted by the differentially expressed proteins in N-PRRSV-infected lungs; C) the GOs targeted by the differentially expressed proteins between N-PRRSV and H-PRRSV infected lungs. The vertical axis is the GO category and the horizontal axis is the enrichment of GO. [file 1743-422X-7-107-S1.TIFF]

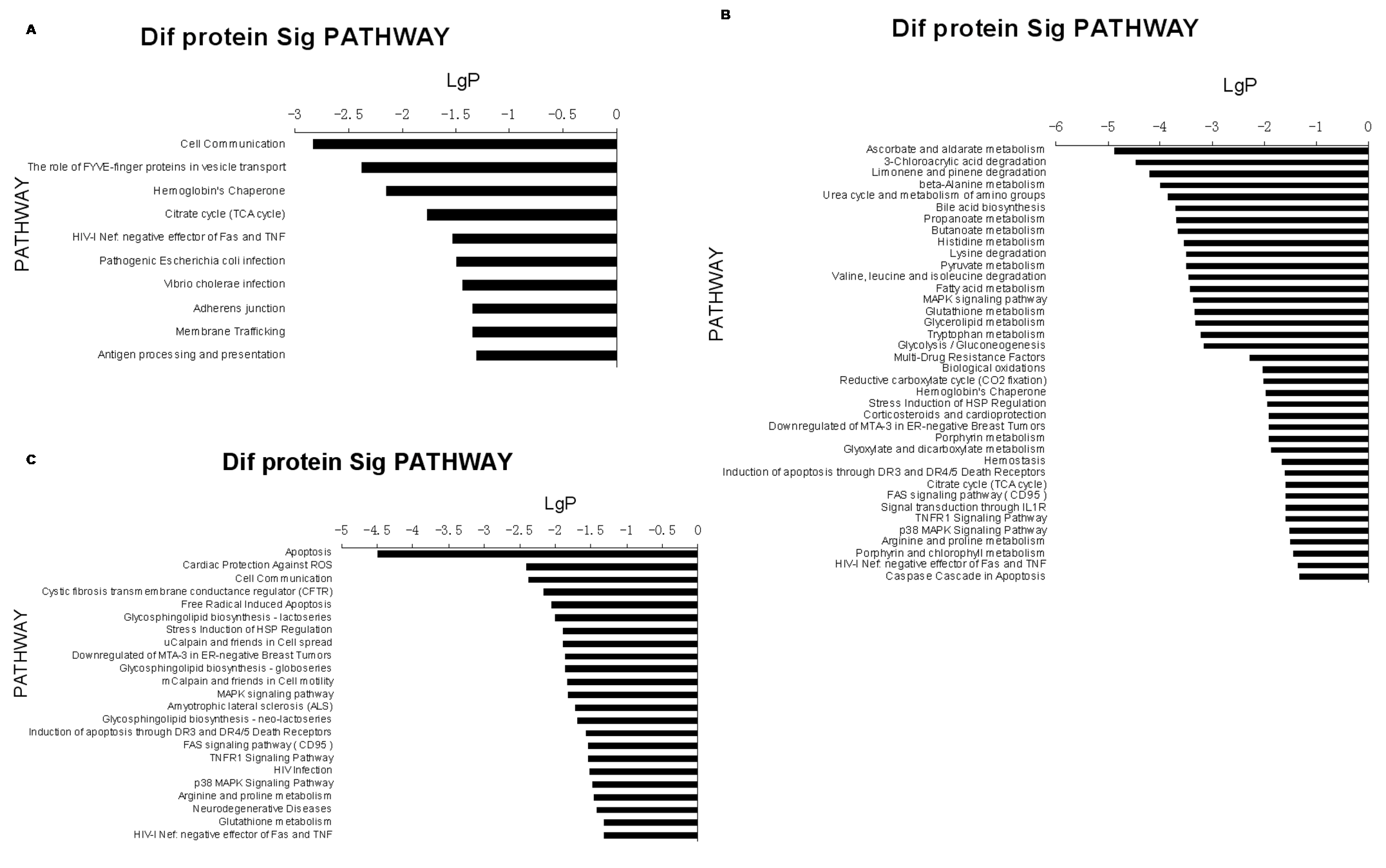

Supplement: Additional file 3 — Signaling pathways of identified proteins. Pathway analysis based on the KEGG, BioCarta, and REATOME bioinformatics database. A P-value of <0.05 and an FDR of <0.05 in the two-side Fisher's exact test were selected as the significant criteria. A) significant signaling pathways of these identified proteins H-PRRSV infected groups; B) significant signaling pathways corresponding to N-PRRSV infected groups proteins; C) significant signaling pathways involved in N-PRRSV versus H-PRRSV infected groups proteins. The vertical axis is the pathway category and the horizontal axis is the lgP(log(p Value)) of these significant pathways. [file 1743-422X-7-107-S3.TIFF]
