# Supplementary material for: Proteome changes of lungs artificially infected with H-PRRSV and N-PRRSV by two-dimensional fluorescence difference gel electrophoresis
Source: Virol J. 2010 May 26;7:107. doi: 10.1186/1743-422X-7-107 (PMC2887434; doi:10.1186/1743-422X-7-107)
Supplement: Additional file 2 — Different expression of proteins after PRRSV infected depend on time points. A) Different expression of proteins between H-PRRSV inoculated lungs and control depend on time points; B) Different expression of proteins between N-PRRSV inoculated lungs and control depend on time points. [file 1743-422X-7-107-S2.DOC]

**Additional file 2** Different expression of proteins after PRRSV infected depend on time points

# A) Different expression of proteins between H-PRRSV inoculated lungs and control depend on time points; A indicates uninfected control, B indicates 96h post H-PRRSV-inoculation, C indicates 168h post H-PRRSV-inoculation;

# B) Different expression of proteins between N-PRRSV inoculated lungs and control depend on time points; A indicates uninfected control, D indicates 96h post N-PRRSV-inoculation, N96, E indicates 168h post N-PRRSV-inoculation.

# A) Different expression of proteins between H-PRRSV inoculated lungs and control depend on time points; A indicates uninfected control, B indicates 96h post H-PRRSV-inoculation, C indicates 168h post H-PRRSV-inoculation.

Master NO: 187

Human protein (Abbr.): ARHGAP29

Master NO: 342

Human protein (Abbr.): TF

Master NO: 352

Human protein (Abbr.): NDUFS1

Master NO: 371

Human protein (Abbr.): HSPA8

Mater NO: 381

Human protein (Abbr.): KRT79

Mater NO: 467

Human protein (Abbr.): STIP1

Mater NO: 507

Human protein (Abbr.): AHSG

Mater NO: 552

Human protein (Abbr.): DPYSL2

Mater NO: 690

Human protein (Abbr.): ACTG1

Mater NO: 882

Human protein (Abbr.): ANXA2

Master NO: 885

Human protein (Abbr.): IDH3A

Mater NO: 1461

Human protein (Abbr.): HBB

# B) Different expression of proteins between N-PRRSV inoculated lungs and control depend on time points; A indicates uninfected control, D indicates 96h post N-PRRSV-inoculation, N96, E indicates 168h post N-PRRSV-inoculation.

Master NO: 242

Human protein (Abbr.): FLNA

Mater NO: 316

Human protein (Abbr.): ACO2

Mater NO: 481

Human protein (Abbr.): LMNA

Mater NO: 612

Human protein (Abbr.): NTF4

Mater NO: 614

Human protein (Abbr.): ALDH2

Mater NO: 616

Human protein (Abbr.): LAP3

Mater NO: 638

Human protein (Abbr.): ALDH9A1

Master NO: 656

Human protein (Abbr.): FLOT1

Mater NO: 666

Human protein (Abbr.): CCDC13

Mater NO: 938

Human protein (Abbr.): ANXA1

Mater NO: 1016

Human protein (Abbr.): FECH

Mater NO: 1038

Human protein (Abbr.): DDAH2

Mater NO: 1173

Human protein (Abbr.): HSPB1

Mater NO: 1200

Human protein (Abbr.): GSTP1

Mater NO: 1250

Human protein (Abbr.): PEBP1

Master NO: 1312

Human protein (Abbr.): TAGLN

Mater NO: 1316

Human protein (Abbr.): CFL1

Master NO: 1491

Human protein (Abbr.): COX5A
